# Multiple stakeholder perspectives of factors influencing differential outcomes for ethnic minority students on health and social care placements: a qualitative exploration

**DOI:** 10.1186/s12909-021-03070-3

**Published:** 2022-01-04

**Authors:** Julie Nightingale, Jackie Parkin, Pete Nelson, Shirley Masterson-Ng, Jacqui Brewster, Temitope Labinjo, Deborah Amoakoh, David Lomas, Ifrah Salih, Deborah Harrop

**Affiliations:** 1 Department of Allied Health Professions, Sheffield Hallam University, Collegiate Campus, Sheffield, S10 2BP UK; 2 Department of Nursing and Midwifery, Sheffield Hallam University, Collegiate Campus, Sheffield, S10 2BP UK; 3 Department of Social Work, Social Care and Community Studies, Sheffield Hallam University, Collegiate Campus, Sheffield, S10 2BP UK; 4 Department of Radiology, King’s College Hospital NHS Foundation Trust, Denmark Hill, London, SE5 9RS UK

**Keywords:** Ethnicity, BAME, BME, Student experience, Clinical placements, Allied health professions, Social work, Nursing, Midwifery

## Abstract

**Background:**

Despite considerable efforts there continues to be a degree awarding gap within the United Kingdom (UK) between the proportion of White British students receiving higher classifications, compared to ethnic minority UK-domiciled students. Practice placement elements constitute approximately 50% of most health and social care programmes, yet surprisingly little research exists related to the factors which may contribute to ethnic minority student placement outcomes or experiences. This study bridges this evidence gap by exploring factors influencing differential placement outcomes of ethnic minority students from the perspectives of key stakeholders.

**Methods:**

The study followed a descriptive qualitative research design and was multi-disciplinary, with participants drawn from across nursing, midwifery, social work and the allied health professions. Participants from four stakeholder categories (ethnic minority students, academic staff, placement educators and student union advisors) were invited to join separate focus groups. Focus groups were recorded and transcribed and analysed thematically.

**Results:**

Ten separate focus groups [*n* = 66] yielded three primary themes: 1) *recognition*, which highlighted stakeholder perceptions of the issues [sub-themes: acknowledging concerns; cultural norms; challenging environments]; 2) *the lived experience*, which primarily captured ethnic minority student perspectives [sub-themes: problematising language and stereotyping, and being treated differently]; 3) *surviving not thriving*, which outlines the consequences of the lived experience [sub-themes: withdrawing mentally, feeling like an alien].

**Conclusion:**

This study presents a rich exploration of the factors affecting differential outcomes of ethnic minority students on practice placements through the lens of four different stakeholder groups. To our knowledge this is the first study in which this comprehensive approach has been taken to enable multiple viewpoints to be accessed across a wide range of health and social care professions. The issues and challenges raised appear to be common to most if not all of these disciplines. This study highlights the urgent need to value and support our ethnic minority students to remove the barriers they face in their practice learning settings. This is a monumental challenge and requires both individuals and organisations to step up and take collective responsibility.

## Introduction

Many countries have been struggling to address the inequity of access to higher education for different ethnic and racial groups. In the United States of America (USA), for example, USA-domiciled Asians over the age of 25 are more likely to have a Bachelor’s degree or higher than the non-Hispanic white population; degree attainment for the Black and Hispanic communities lags behind significantly [1, 2]. Facilitating access to university is, however, not enough; ethnic and racial differences prevail in both degree completion (attainment) and degree classification (degree awarding gap).

In the United Kingdom (UK), white British students are more likely to receive higher degree classifications compared to ethnic minority UK-domiciled students. Outcomes vary considerably by ethnic group, with a much smaller awarding gap between White and Chinese students compared to the gap between White and Black students [3]. Similar ethnicity awarding and attainment gaps have also been reported in the Netherlands, USA and Australia [4]. Educational interventions to reduce ethnicity attainment and awarding differentials are invariably focused to campus-based solutions, whereas the influence of professional practice outcomes on degree attainment and awarding gaps is rarely investigated. Some data is available that confirms that attainment and awarding differentials are associated with professional practice elements of health and social care programmes. In one institution, physiotherapy students from ethnic minority backgrounds were found to receive statistically significantly lower marks than white peers in final clinical placements, although the difference was small [5]. In a larger study analysing data from four institutions in England (*n* = 1851), Norris et al. highlighted significantly lower assessment scores for minority ethnic groups compared to White British students, most notably in clinical and observed assessments [6].

However no studies directly investigate the factors influencing the placement awarding gaps or attainment differentials and, in UK literature, the experiences of visiting international students rather than UK-domiciled minority ethnic students are commonly presented. However a few research studies explore the practice placement experiences of minority ethnic students in health and social care, and several themes can be drawn from this small body of research.

Students often describe the university as a ‘safe haven’ and find the transition to a less regulated placement environment as stressful [7]; strong tutor-student relationships in both academic and placement environments are contributors to success [8–10]. Indirect or subtle racism from service users, academics, peers and practice placement staff affects many ethnic minority students across all aspects of their lives, not only on placement [8–11]. Generalizations are often made about a student’s culture and identity based on the colour of their skin [8, 9, 11–13]. Labelling and stereotyping based on physical appearance, body language and mannerisms, culture, and accents/dialects/idioms are also recurring themes [8, 9, 11]. Many encounters negatively impact on student experience and confidence by increasing isolation [12] and feelings of ‘being judged’ [8, 9, 11] and ‘being different’ [7–11], in some instances affecting learning experiences and outcomes [13].

Coping strategies outlined by students include adopting a positive attitude towards studies and their cultural background [10], using self- protective measures and confronting any areas of concern directly [11]. Recommended interventions include tailored education for placement staff [9] regarding concepts such as white privilege and its impact [11], and a requirement for staff to review their programmes to ensure that diversity and culture is explicitly valued [10, 12] and that ethnic minority students are not marginalised [7, 13]. Formal agreements between universities and placement providers should include anti-racist standards for placement [9] alongside more comprehensive reporting and monitoring mechanisms [8].

In summary, there is a paucity of research focusing upon the factors affecting ethnic minority student attainment / awarding differentials and their experience on practice placements. This is surprising given that the professional practice elements constitute approximately 50% of most health and social care programmes. The evidence base is weak; most studies are either single-centre related to a single programme of study, or exploring a single profession and/or a single ethnic group. As wide differences in educational systems, models of clinical training and cultural issues occur internationally, the context and transferability of the research is highly variable. There is clearly an evidence gap in our understanding of the factors influencing ethnic minority student attainment differentials and awarding gaps in health and social care professional practice placements. This study aims to bridge this evidence gap.

## Methods

The aim of the study was to explore, from the perspectives of key stakeholders, the range of factors which may contribute to a potential ethnicity attainment differential / awarding gap on health and social care practice placements. While the study was based at a single institution, the research was multi-disciplinary, with both researchers and participants drawn from across nursing, midwifery, social work and the allied health professions. The study followed a descriptive qualitative research design [14] which employed focus groups as the data collection method. According to Bradshaw et al. [14], qualitative descriptive research studies seek to explore and understand a little known process or phenomenon, or the perspectives of those involved, “providing a rich description of the experience depicted in easily understood language” (p3).

### The research team

The research team consisted of academic staff and student researchers from a mix of ethnicities, genders, and professional backgrounds relevant to the research context (Table 1). Research experience varied across the team with experienced researchers pairing up with students and early career or novice researchers at various stages of the study.

**Table 1:** Research team characteristics

Researcher	Gender	Ethnicity	Professional background	Research experience
1	F	White	DR	Experienced researcher
2	F	White	N	Early career researcher
3	M	White	SW	Research leader
4	F	White	LDN	Novice researcher
5	M	White	PT	Novice researcher
6	F	Asian	OT	Early career researcher
7	F	Black	N	Early career researcher
8	F	White	IS	Research leader
9	F	Black	DR	BSc student researcher
10	F	Black	N	PhD student researcher

Key: *DR* diagnostic radiography, *IS* information specialist, *LDN* learning disability nursing, *N* nursing, *OT* occupational therapy, *PT* physiotherapy, *SW* social work

### Recruitment and data collection

Following institutional ethical approval, participants were invited to join separate focus groups for each stakeholder category (Table 2). Student participants were studying on pre-registration health or social care courses, and identified themselves as a UK-domiciled person from an ethnic minority background. At the time of data collection the term BAME (Black, Asian and Minority Ethnic) was used to identify these students, but in the light of subsequent debate and scholarship the term ethnic minority is used throughout this paper. These student participants were drawn from all years of study but they had to have experienced at least two practice placements situated in the wide geographical region serviced by the institution. Placement types, location and duration were variable across the different professional programmes, and included placements in acute and community settings, rural and urban locations, and placements in wards, hospital departments or social care and third sector placements. A variety of methods were used to recruit student participants, including advertising via social media, course leader announcements and on-line learning platforms.

**Table 2:** Stakeholder focus group characteristics

Stakeholder group	No. focus groups	No. participants	Ethnicity	Professional groups represented
Ethnic minority students	5	24	Black (11) White (2) Asian (4) Ethnicity not stated) (7)	SW (3); N (3); LDN(4); OT (6); PT (1) DR (6); TR (1)
Academic Staff (programme leads and placement coordinators)	5	22	White (21) Asian (1)	DR (3); TR (1); N (9); ODP (1); SW (4); M (1); Biochemistry (1); Cross Faculty (2)
Placement Educators	2	16	White (16)	OT (5); PT (2); N (9)
Student’s Union Advisors	1	4	White (4)	N/A
**Total**	13	66		

Key: *SW* social work, *OT* occupational therapy, *PT* physiotherapy, *TR* therapeutic radiography, *DR* diagnostic radiography, *LDN* learning disability nursing, *N* nursing, *M* midwifery, *ODP* operating department practice

Staff participants were invited via email distribution list invitations to stakeholders who had experience of supporting ethnic minority students with their placement practice. These stakeholders included academic staff with overall responsibility for placement education (programme leaders and placement coordinators), agency based placement educators and student union advisors. Table 2 highlights that the ethnicity of the staff participants is overwhelming White. The staff participants were invited for their experience of working with students on placements, or of roles with responsibility for placement learning or student support. There was no direct attempt to recruit staff from ethnic minority backgrounds, as this lack of ethnic diversity reflects the academic and placement supervisor community at the time of the study.

**Fig. 1:**
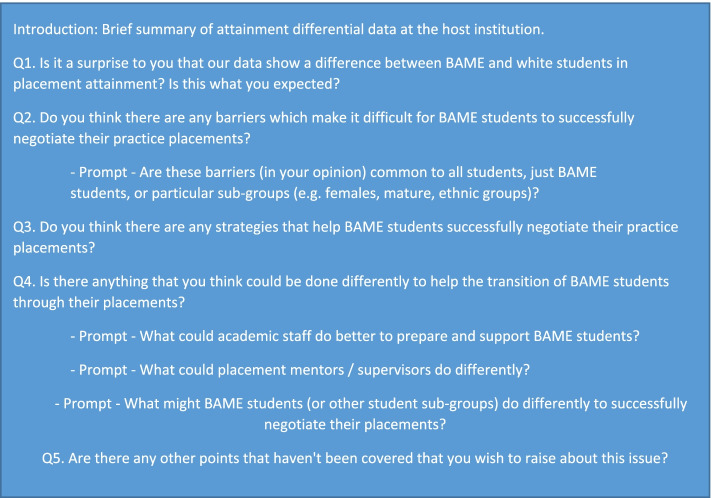
Focus group question schedule for all groups

While semi-structured interviews are often deployed to investigate sensitive topics, focus groups were selected to enable a ‘safe space’ to be created for collective voices to be shared and heard, giving confidence to some participants who might otherwise have been reluctant to come forwards. Following written informed consent, each focus group was delivered by two facilitators and was with the participants’ permission audio recorded. During the focus group one researcher was assigned to ask the questions, the second facilitator assisted with prompts and recorded anything of note observed throughout the focus group. This included any emerging thoughts, and notes on body language and dynamics of the group. The focus groups all followed the same question schedule [14] (Fig. 1), with facilitators able to subtly alter the wording, prompts or order of questions as appropriate to their group.

### Data analysis

The data generated from the focus groups was analysed using a structured framework as recommended in descriptive qualitative research [14]; the six stages proposed by Braun and Clarke were used [15]. 1) *Familiarise yourself with the data.* The focus groups were transcribed by a professional transcriber. Transcripts were proof read for accuracy, alongside listening to the audio file. One researcher read the transcript, noting any initial thoughts. 2) *Generating initial codes.* Following several re-readings of the transcript preliminary codes were assigned to the data to describe all areas of interest. 3) *Searching for themes***.** At this stage in the analysis process codes were searched for patterns and then grouped into potential themes. 4) *Reviewing the themes.* Potential themes were reviewed and discussed with a second researcher. The themes were compared across transcripts within each stakeholder group and some refining and reorganising took place at this stage to ensure that the themes were appropriate to the data set as a whole (comparing across stakeholder groups). 5) *Defining and naming the themes.* Once the final themes were agreed these were shared with the wider research team for further discussion, refinement, and validation. 6) *Producing the report.* Three primary themes, encompassing eight subthemes were identified.

### Rigour

Four broad principles first described by Yardley (2000) have been applied for guiding and subsequently evaluating this qualitative study [16]. *Sensitivity to context* has been achieved through the use of verbatim quotes to give the participants a voice. However some of the researchers involved in this study are academics from white, middle class backgrounds. Their ‘insider-outsider’ status and related power dynamics [17] may have influenced the ways in which some students responded to questions. This effect was reduced by using focus groups which enabled participants to gain confidence from the support of fellow participants, as well as ensuring that researchers who were course leaders did not question students from their own course. *Commitment and rigour* have been demonstrated by the undertaking of a thorough analysis of the data and checking of interpretations across the researcher team. While the findings of this study cannot claim to be representative of all of the factors influencing ethnic minority students’ placement experiences and attainment differentials, the student participants have been afforded the opportunity to provide new appreciations of their lived experiences and thereby reveal valuable insights. *Transparency and coherence* have been achieved by carefully outlining each stage of the research process. Finally, *impact and importance* has been demonstrated by this article beginning a conversation related to the under-researched and under-recognised area of ethnic minority placement attainment differentials and students’ experiences of practice placement.

## Results

**Fig. 2:**
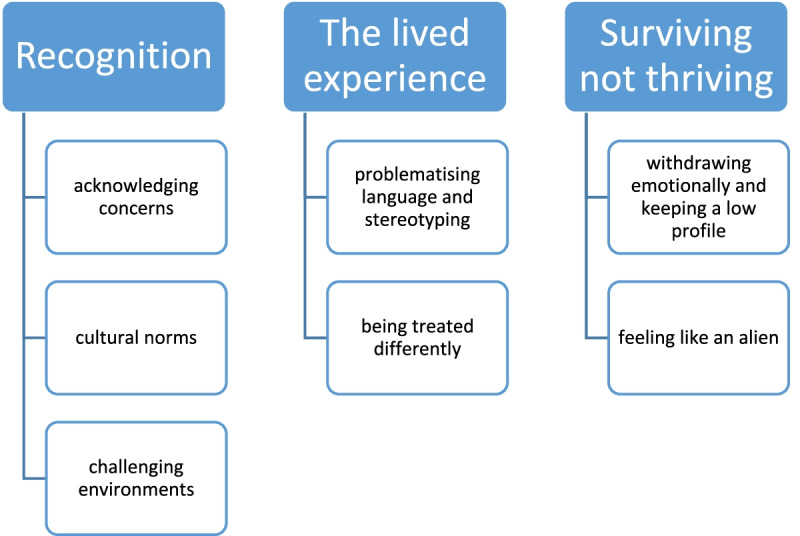
Schematic diagram of the three themes and seven sub-themes

Thirteen focus groups were delivered as shown in Table 2, involving a total of 66 participants. Three primary themes were identified (Fig. 2). The first theme *recognition* highlighted primarily staff stakeholder perceptions of the issues within three sub-themes: acknowledging concerns; cultural norms and challenging environments. The second theme *the lived experience* captured primarily the ethnic minority student perspectives, housed within two sub-themes: problematising language and stereotyping, and being treated differently. The final theme *surviving not thriving* outlines the consequences of the lived experience housed within two sub-themes: withdrawing emotionally and keeping a low profile, and feeling like an alien.

### Theme 1: recognition

*Acknowledging concerns* related to recognition by many participants of the concept and reality of ethnic minority degree awarding gaps. Awareness of pre-registration fitness for practice ethnicity differentials were highlighted strongly in some participant groups (students, academics, student union advisors), but less so in placement educator groups who may be unfamiliar with university data. Academic staff discussed ‘behaviours’ which had been highlighted in recent ethnic minority students fitness for practice referrals or placement fails, included timekeeping, reliability, not following processes, abrupt or confrontational manner, and poor communication. The academic staff were unsure whether this data was an indication of underlying racism in some placements, or whether there were aspects of placements that were impacting negatively on student behaviour.

*"what made me concerned was that I was doing very, very similar referrals for almost identical students" [Academic]*

*"...out of the last probably six [referrals], I would say probably five would be BME and it is a concern" [Academic]*

Staff participants discussed the influences of *cultural norms* and challenges that may be faced by some students, highlighting that some cultures had the characteristics of high expectations of success, of internalising problems and not sharing issues; of over-respect for authority and in some cultures a degree of lack of respect for women. Some students recognised cultural norms which have been shaped by experiences:

*"From a cultural perspective it’s often frowned upon for them [ethnic minority students] to...question supervisors" [Academic]*

*"Two or three male students have struggled to be assessed by female mentors" [Placement educator]*

*"Obviously because we are from the black minority our self-esteem might be really low according to what we’ve had before, had from different corners, so I think we just need a boost, reassuring". [Student]*

*"I feel like we are from a cultural background that you have grown up telling you don’t tell people everything about yourself. I might have a big problem… But most African backgrounds I think you are told you just don’t expose yourself unless it’s right and necessary". [Student]*

Cultural norms also related to the inherent culture that develops within a profession. Health and social care students were expected to conform to a traditional professional framework and ‘way of being’, but for some ethnic minority students this meant conforming to ‘white’ culture as well. Placement educators acknowledged that the student profile was changing but perhaps the educational model was now outdated:

*"I think … about the way that they [ethnic minority students] express their emotions. I think we’re not used to dealing with that either. So perhaps we don’t handle it as well as we could, because we’re used to that stifling of emotions, aren’t we?" [Placement educator]*

*"I wonder if in maturing professionally they just have a different journey personally to other students, but it may be that their mentors expect a different reaction." [Placement educator]*

Academic and student union staff strongly expressed the view that cultural norms are less important than the *‘back story’*, which is often hidden from view, yet explains and often justifies behaviours:

*"There are some really harrowing, harrowing stories...They don’t want to talk to you about it. It’s really hard for them, because they’ve kind of dealt with that part of their life and tried really hard to move on". [Student Union Advisor]*

*Challenging environments* describes the wide range of placements in health and social care that ethnic minority students may be exposed to in their training. Examples were given where placement geography resulted in variation in placement experiences, particularly where students were working in *‘overwhelmingly white’* communities.

*I don’t want to stereotype either, but with placements in the rural areas, I think most of the placements in the rural areas have a problem when it comes to black minority. [Student]*

Students’ union advisors were concerned that they were hearing about the same ‘*repeat offenders*’ over time, yet students were reluctant to take their concerns to their tutors. This *‘once-only disclosure’* from the students put student union staff in a difficult position as they had spoken to students on a confidential basis, thus perpetuating the issues raised. Staff referred to these brave disclosures as *‘the tip of the iceberg’*:

*"People are internalising it...they absorb it. They come to us as a confidential service and they will disclose it and then might just never say it again to anyone else". [Student Union Advisor]*

Academic participants outlined a number of particularly challenging settings for some ethnic minority students, including those requiring rapid communication challenges such as fast-paced clinics. Some academic staff and placement educators suggested that some ethnic minority students perform better in one-to-one settings and in professions where they had support from a single mentor. Supervision models where a wide range of professionals support a group of students (occurring in some ward placements, or service departments such as radiology or the operating theatres), were noted to be more challenging for some ethnic minority students. This observation referred to UK-domiciled ethnic minority students, and may be a result of accents, referred to in Theme 2. However students recounted both good and poor experiences in both one-to-one and group supervision settings; the culture of the placement appears more influential than the mode of supervision:

*"I’d certainly say my first placement was very different. It was good, some staff members were good, but there were a couple that were making comments that I didn’t like very much, racist comments, but certainly here on my second placement it’s a completely different culture. Everyone is completely different… every single person in the department I’d say are amazing and I get treated equally and fairly." [Student]*

Student participants tended to focus their focus group conversations on the role of supervisors, mentors and placement staff in assisting their journey through the placement, rather than concerning themselves with challenging situations working with patients and clients. Academics and placement educators, however, highlighted a number of challenging patient and client groups who were more likely to create uncomfortable situations for ethnic minority students. These included the elderly, those with cognitive impairments, patients in the emergency department under the influence of drugs or alcohol and patients within high stress environments such as the operating theatre.

*"He was working in an elderly area and the patient referred to him as a 'man from the colonies' ... none of the staff challenged that or checked that he was OK and it was just accepted ..." [Academic]*

### Theme 2: the lived experience

*Problematising language and stereotyping* was a sub-theme which cut across most participant groups, but was most strongly articulated in the student groups. Stereotyping often began before the student commenced the placement, with their names being the first hurdle to overcome:

*"And so it’s already the name is a barrier, because ...everyone is thinking how we are going to say that, how are we going to pronounce that?" [Student]*

*I have experienced that kind of thing where people don’t look beyond your skin. [Student]*

Academic staff also recognised the unfairness of stereotyping of ethnic minority students by a minority of placement staff:

*"[they] felt that they were being unfairly treated based on the colour of their skin". [Academic]*

*"she said, 'we really struggle with these types of students'." [Academic]*

*Problematising language* described a range of situations in which emphasis was placed on negative attributes at the expense of the positive influences that ethnic minority students can bring in terms of diversity. Accents were a recurring issue described within the student groups, and recognised by most academic staff:

*"I was being told off of my accent and she said that I’ve got a very strong Zimbabwean accent, I need to improve on that… But as a human being you feel down if someone is telling you off about your accent, because you don’t know how to present yourself any more" [Student]*

*"it was sad that my supervisor dwelled more on my accent and she laughed about it." [Student]*

*"she mocked how he spoke ...and I thought well if you’re doing that when I’m here, what are you doing when this poor student’s there on their own..." [Academic]*

This was more than simply their accent being the issue; when some students became stressed the pitch of their voices would rise and this would be mistakenly seen as shouting or aggression. One student was embarrassed when a supervisor questioned her ‘tone’ and asked if English was her first language; she had lived in the UK all of her life:

*"Yeah, because I speak, to me I speak English. I was on the phone … I was like oh my god, do I have tone to my voice, do I sound different, am I different? …I got so embarrassed, I was so embarrassed. I just went quiet and I just like, I was in shock more than anything, so I felt I couldn’t say anything at that time." [Student]*

For some students, challenges with language and accents resulted in miscommunication, misinterpretation and mixed messages. Students had misunderstood jokes and humour in some settings, or their humour has been misunderstood, which was intimidating and isolating. In particular local dialect and colloquialisms caused confusion. Students recounted deploying a ‘fake’ laugh or pretending that they don’t hear; unfortunately, they noted that this makes them appear *‘aloof’*, or *‘slow’.* However, some academic and placement staff empathised with students who may be thinking, writing and speaking often in another language:

*"…she said her first language was her tribe language, second was French, third was Italian and she was writing in her fourth language which was English" [Academic]*

*"… they’re studying and working in a second/third/fourth language and then to add to that we give them medical and nursing terminology." [Academic]*

The majority of the ethnic minority student participants described their lived experience as *‘being treated differently’* to their White student counterparts. While there were some comments related to discrimination in relation to academic settings (most students described the university campus as a ‘safe space’), there were a number of examples of *perceived discrimination* in practice settings, particularly regarding male (often mature) Black students:

*"...quite a lot of students tell us that they feel that they’re being prejudiced against on placement in particular. And it tends to be cultural reasons and it tends to be the students that we see are of a certain age, a certain background". [Student Union Advisor]*

*"We had this one [ethnic minority] lad and they just made him into a laughing stock really. And they made him do every [case] just to get a kick out of it really and just to laugh at him, and they were like setting him up to fail". [Student]*

*'If other students ask (for help) there was no problem, but if I ask it they were taking me as if I’m not understanding what I’m saying ..." [Student]*

Students also identified a general lack of respect and professionalism from some placement staff:

*"where [staff] have been talking and there’s a student in the room ... and they are BME and that they’re not good. But no form of support offered." [Student]*

*"[I was] spoken to like a child" [Student]*

However the day to day lived experience recounted by several students was more of subtle ‘undertone’ rather than overt racism or discrimination. ‘*Active Avoidance’* described the scenarios where students feel alienated by supervisors who avoid working with them or engaging in any “chit chat” between patient/client encounters:

*"...so when they see a [white] student having a chat and having a laugh with staff, and they are not welcomed in the same way..." [Academic]*

*"And you will want to [get involved] but they don’t seem interested ...because they don’t talk that much with you". [Student]*

*"It’s sort of bullying by exclusion, isn’t it, not including them". [Academic]*

Students in this study, even where they had generally positive experiences on placement, all believed that as ethnic minority students they were *‘held to a different standard’* and had to work hard to prove themselves. Academic staff also recognised this in relation to many of the ethnic minority students they had mentored*:*

*"But I think they’ve got double to prove... to prove themselves twice". [Academic]*

*"I feel like I have to do twice as much as the 'locals' to prove myself". [Student]*

*"Maybe I’m just generalising that we [ethnic minority students] all have to work ten times harder because of all these stereotypes and stigmas against us". [Student]*

### Theme 3: surviving not thriving

Students who are experiencing a challenging placement for the reasons previously outlined put in place a number of self-protection measures to reduce their own discomfort and prevent escalation of their worries. *Withdrawing emotionally and keeping a low profile* describes the self-protection measures that some put in place even before commencing the placement; they anticipate prejudice, based on previous life experiences. This potentially shapes their future placement experiences:

*"I would think the stereotyping and discriminative attitudes of people wherever we go will shape our psychological outlook. It will impact on our confidence and low self-esteem. And that is a very, very bad barrier… I don’t think in future I would go and will discuss or challenge you wherever I feel things are not going well, I will stay in the corner and always go with the flow. So for me it’s been things around people’s attitudes which have shaped the way I feel about myself." [Student]*

A sense of the coping strategies used to protect students is captured in phrases repeated consistently across the different focus groups, and by students from diverse ethnic backgrounds and different professional groups. These include: *“don’t rock the boat”; “keep your head down”; “take it on the chin.”*

Some students subsequently encountered very positive placements which helped them to re-evaluate their perceptions and build their confidence. However where students’ fears were realised, the consequences resulted in them *‘feeling like an alien’*, an ‘outsider’ exhibiting a high degree of suspicion and mistrust around colleagues. One student explained the impact of placement staff flippantly discussed a neighbouring community as being *‘OK apart from the bloody Asians’*:

*"...you just feel like I’ll just ignore that, I didn’t hear that. But at the same time in the back of your mind you do feel a bit alien. You don’t know who’s on board with these opinions and who is not on board: who’s against you or not against you." [Student]*

Students in such situations became adept at concealing concerns and were resilient in adversity:

*"Sometimes it’s a bit of a headache just to challenge it. I’m like do you know what, I just want to pass. I don’t want to cause anyone any trouble. I just get on with it … Because I’d rather fake laugh, get on and do my work, than go and do a whole report and it feel really awkward afterwards. But I know I should challenge them…" [Student]*

*"You’ve got to fake it to make it...it’s just smiling and gritting your teeth because you have to do it because you don’t fit in, but you’ve got to get through, so you have to do it". [Student]*

## Discussion

Most staff participants in this study recognised a differential in ethnic minority student placement attainment and professional ‘Fitness for Practice’ referrals, also identified by Naylor et al. 2013 [5] and Fairtlough et al. [8], where ethnic minority students appeared more vulnerable to be referred, fail or withdrawn from placements. Academic staff could articulate issues that may impact on the placement experiences and outcomes of ethnic minority students, and were keen to *‘make a difference’*. However being predominantly of White ethnicity their lived experiences were potentially very different to the students that they sought to help. The placement educators, also from White ethnic backgrounds, were drawn mainly from physiotherapy and occupational therapy professions which experience lower ethnic minority representation than some other health and social care professions; indeed physiotherapy in the UK has been previously described as a ‘white profession’ [7]. In some other professions such as nursing there is a mismatch between the profiles of students and academic staff [17]; this white dominance strengthens the power imbalance between student and educator. The much debated term ‘white privilege’ may be applied in these healthcare education settings. This concept acknowledges that some White communities do suffer disadvantage and deprivation, but the colour of their skin has not been a factor in life chances as it is likely to have been in many ethnic minority communities [17] due to societal, economic or political factors. While the White ethnicity of the staff participants in our study may be seen as a limitation it is an accurate representation of the local setting. Academic staff voiced their keenness to work with ethnic minority students to co-produce strategies to improve placement experiences, and student union advisors similarly were pleased to have a forum in which to anonymously share the experiences recounted to them by ethnic minority students and to co-design improved reporting mechanisms for raising concerns.

However for these strategies to be successful, a thorough understanding of the factors affecting placement attainment and of the lived experience and coping strategies of ethnic minority students was needed. This is the first qualitative study to explore the factors influencing placement attainment, awarding gaps and experiences across a range of health and social care professions. Previous studies have focused mainly on social work student experiences [8–11, 13], physiotherapy students [5–7, 18] or have primarily studied campus-based experiences [6]. Few studies have focused on health courses and while one paper focused on (physiotherapy) placement experiences [7], it only involved three ethnic minority participants. The current study provides a unique and important insight to begin to fill this knowledge void.

The three themes presented in this study, namely *‘recognition*’, ‘*the lived experience*’ and ‘*surviving not thriving*’, clearly demonstrate parallels to several studies centred on social work education [8, 10, 11, 13]. In particular, stereotyping was a recurring theme across these studies; our student participants believed that it began even before the placement when supervisors were introduced to names with challenging pronunciations. Accents were often ‘problematised’ as in previous studies [8, 9]; and some ethnic minority students were laughed at for their ‘strange’ pronunciation as reported also by Hillen and Levy [10]. Indeed Hollindrake et al. postulated that students were seen as ‘less capable’ because of their accents and this makes them feel like ‘outsiders’ [11]. Our students also explained how their accents raised the potential for miscommunication and misinterpretation, resulting in students deploying a self-protection strategy such as ‘hanging back’ and keeping themselves on the periphery of any placement group. In other words they found themselves adopting an ‘outsider’ position, with a feeling of ‘not belonging’ as described by Hughes [18].

Most students in this study described the university setting as a ‘safe space’, not dissimilar to the ‘safe haven’ described by physiotherapy students in a study by Cassidy et al. [7]. In contrast practice placements were seen as unpredictable and with a higher potential for overt racism to be experienced, particularly from some vulnerable client groups. Staff participants recognised settings where this may be more likely to occur; settings with higher risks for racism have also been described in social work [8]. However the most stressful aspect for students was anticipating how staff would respond to these incidents. Ignoring the issue or ‘making a scene’ was likely to make the student more anxious; they wanted the incident to be acknowledged and to be asked if they were alright. The lack of this follow up in some instances by clinical colleagues raises concerns, especially within these ‘caring’ professions.

For most participants, awareness of an ‘undertone’ of more covert racism was of greater concern, resulting in them ‘being treated differently’. This concept was commonly described across many social work studies [8–11]. An example of this concept in our study was the widely held perception that ethnic minority students appeared to be ‘held to a different standard’, also identified by other authors. Fairtlough et al. noted that even where ethnic minority students had positive placement experiences they still felt that the expectations from them were higher [8], with Thomas et al. describing this scenario as a feeling of ‘being watched’ [9]. Hughes et al. also related similar concepts of having to work harder, with students having to over-achieve to be seen as competent [18].

Our participants described the ‘active avoidance’ of ethnic minority students by some staff, for example not engaging in ‘small talk’ between clients as they witnessed occurring with white students. This resulted in increasing isolation and a lack of belonging [8, 9], leading to anxiety of sharing information about themselves or their culture. Hollindrake et al. had also identified a gradual ‘withdrawal’ of cultural sharing [11]; lack of acknowledgement of culture may result in or from a ‘colour-blind approach’ [9] which reduces the potential for a rich cultural diversity in placement settings. Viewing ethnic minority students from a strengths-based cultural wealth perspective may help to oppose the more pervasive ‘deficit model’ which aligns with social and racial justice [9, 15, 19]. Yosso’s model [19] encompasses six forms of cultural wealth which may help to guide future placement strategies; aspirational, linguistic, familial, social, navigational, and resistance. Nurturing community cultural wealth emphasises the strengths that ethnic minority students inherently possess as identified in social work by Sangha [13].

The consequence of subtle, yet repetitive, experiences of being treated differently on practice placement was that students adopted self-protective mechanisms; students describe ‘feeling like an alien’, causing them to deploy strategies such as ‘withdrawing emotionally’ and ‘keeping a low profile’. As recounted in a study by Hammond et al. [12] which focusses on the wider curriculum, it was easier to not make a fuss and allow incidents to pass; Beagan argues this is an effective self-preservation mechanism as students can be perceived as too sensitive by the privileged majority who are ignorant to the issues faced [20]. These mechanisms become learned behaviours over time [21], but they are potentially damaging strategies in terms of students’ longer term professional experiences and attainment. We have described this concept as ‘surviving not thriving’. A phrase often used in wellbeing and life coaching, it has also been applied to settings where individuals are transitioning through career stages, including early career teacher resilience [22], student midwife transition to practice [23] and the experiences of motherhood in early career researchers [24]. While many ethnic minority students in our study showed a persistence to push through to the end of their course, the practice placements should be an opportunity for them to prosper and flourish as they consolidate their academic learning into practice. Of serious concern is that so many students recounted similar experiences and shared commonly held fears and concerns related to placements. The majority of these students, drawn from different ethnic groups, different health and social care professions, and having experienced a range of unique placements across a wide geographical region, raised similar issues which were commensurate with ‘surviving not thriving’ [22–24]. Participation in the focus groups was optional and therefore students are self-selecting; it is possible that there is selection bias with only those with poor experiences wishing to articulate their concerns. Some students may have been dissuaded from participating due to the perceived consequences of speaking up and the power imbalances between staff and students. However many of the students recounted good as well as poor placement experiences, and they expressed their appreciation of placement educators who had supported them in a positive way. This positive learning experience had made them realise that they, and other students following them on their journey, should be able to expect more, and this is why they wanted to take part in the study. Two of the student participants were White women who had themselves experienced only positive placements, yet they requested to participate to share their concerns regarding the differential treatment of some of their fellow ethnic minority students. This is an example of ‘ally-ship’ in action; racial justice allies are White students who actively work against the system of oppression that maintains their power [25]. They and the staff participants believed that change was needed to enable all students to be supported to thrive, yet Hammond et al. warns that gains may be minimal if the white dominant narrative remains unchallenged [12]; educators rather than ethnic minority students need to challenge their own and institutional practices. However educators in our study often lacked the confidence to challenge; by not questioning and challenging practices, Beagan asserts that educators are complicit in enabling marginalisation and inequalities [20]. This suggests that there is a vital and urgent training need for staff across both academic and clinical settings, and regulatory and professional bodies must take a lead role in driving social justice changes through their expectations of practice and education.

In drawing our conclusions we are conscious that this is a UK based study drawing on a sample from a specific geographical area and underpinned primarily by UK literature and as such it prohibits generalisation. There is a wide range of international literature exploring the ethnic minority educational experience, including the USA [26], Canada [18] and Australia [27]; many findings of which our study confirms. We are aware however that the experience of racialised groups in the UK has a specificity stemming from colonial power and the lack of an ethnic minority indigenous population that merits individual focus. Learning from international studies remains important, alongside a recognition of difference, if improvements are to be made and if the finding from Canada by Hughes et al. [18], that the current state of institutional racism within a profession engenders a perception of hopelessness and acceptance in students, is to change.

## Conclusion

This study presents a rich exploration of the potential factors influencing differential attainment and awarding gaps of ethnic minority students on practice placements, undertaken through the lens of four different stakeholder groups (students, academics, placement educators and student union advisors). To our knowledge this is the first study in which this comprehensive approach has been taken to enable multiple viewpoints to be accessed across a wide range of health and social care professions.

The issues raised appear to be common to most if not all of these disciplines, but as the placement educational and supervision models differ from one profession to another, the checks and balances (moderators) may also be unique to each professional setting. This makes providing effective solutions challenging, but this qualitative research is the first step in opening conversations with placement educators across multiple settings. In our experience sharing the student narratives have resulted in a degree of shock and occasional denial, followed by a firm commitment to co-develop strategies to improve the placement experiences of ethnic minority students. All stakeholders had suggestions for improvements which may provide ethnic minority students with a greater sense of belonging in their health and social care placements.

Recommendations arising from this study focus on promoting cultural change and improving systems and processes. Cultural changes include:

- Raising awareness of the lived experience of students from racially minoritised students on practice placement to all placement partners;
- Providing anti-racism training for all academic and clinical colleagues involved with student placements. This should go beyond standard ‘unconscious bias’ training to include more nuanced ‘active bystander’ training;
- Placement preparation sessions for students should highlight the potential for structural racism in placement settings and the potential for racial incidents. They should explicitly identify support and reporting mechanisms and the power of allyship;
- Academic and placement leaders to move away from the deficit model of addressing issues with ethnic minority students to a strengths-based approach of focusing on what diversity can bring to the professions. This may be enhanced by institutional support for role models and champions for the racially minoritised student community.

The required changes to processes include:

- Creation of and sign-posting to clear and explicit processes for reporting racial incidents in relation to placement;
- Collaboration between Higher Education Institutions and their placement partners to address any concerns raised by students, and to provide independent support to students to address the low rate of reporting of placement issues (potentially due to the unequal power differential between student and supervisor).
- Development of processes and strategies to increase awareness of systemic racism and empower staff to change these systems

This study highlights the need to value and support our ethnic minority students to remove the barriers they face in their practice learning. This is a monumental challenge and requires both individuals and organisations to step up and take collective responsibility. We cannot delay tackling these issues any further.

## Data Availability

Data analysed during the current study are available from the corresponding author on reasonable request.

## Abbreviation

BAME: Black, Asian and Minority Ethnic
